# Potential of biosynthesized silver nanoparticles using *Stenotrophomonas* sp. BHU-S7 (MTCC 5978) for management of soil-borne and foliar phytopathogens

**DOI:** 10.1038/srep45154

**Published:** 2017-03-27

**Authors:** Sandhya Mishra, Braj Raj Singh, Alim H. Naqvi, H. B. Singh

**Affiliations:** 1 Department of Mycology and Plant Pathology, Institute of Agricultural Sciences, Banaras Hindu University, Varanasi, 221005, India; 2 Centre of Excellence in Materials Science (Nanomaterials), Department of Applied Physics, Z. H. College of Engineering and Technology, Aligarh Muslim University, Aligarh, India

## Abstract

*Stenotrophomonas* sp. is emerging as a popular microbe of global concern with various potential ecological roles. Biosynthesis of gold and silver nanoparticles (AgNPs) using this bacterial strain has shown promising applications in life sciences. However, there is no report on efficient agricultural applications of biosynthesized AgNPs using *Stenotrophomonas* sp. In this regard, successful biosynthesis of AgNPs using *Stenotrophomonas* sp. BHU-S7 (MTCC 5978) was monitored by Uv-visible spectrum showing surface plasmon resonance (SPR) peak at 440 nm. The biosynthesized AgNPs were spherical with an average mean size of ~12 nm. The antifungal efficacy of biosynthesized AgNPs against foliar and soil-borne phytopathogens was observed. The inhibitory impact of AgNPs (2, 4, 10 μg/ml) on conidial germination was recorded under *in vitro* conditions. Interestingly, sclerotia of *Sclerotium rolfsii* exposed to AgNPs failed to germinate on PDA medium and in soil system. Moreover, AgNPs treatment successfully managed collar rot of chickpea caused by *S. rolfsii* under greenhouse conditions. The reduced sclerotia germination, phenolic acids induction, altered lignification and H_2_O_2_ production was observed to be the probable mechanisms providing protection to chickpea against *S. rolfsii*. Our data revealed that AgNPs treated plants are better equipped to cope with pathogen challenge pointing towards their robust applications in plant disease management.

The genus *Stenotrophomonas*, previously named as *Pseudomonas maltophilia*, phylogenetically belongs to γ-subclass of Proteobacteria^1^,^2^. This novel genus was first proposed by Palleroni and Bradbury^3^ after studying the notable differences between *P. maltophilia* and closely associated xanthomonads group. Later, on the basis of heterogeneity on the phenotypic and genotypic level, seven species of *Stenotrophomonas* has been recognized so far between 1993 and 2007^4^. This ubiquitous microorganism is found in a broad range of ecological niches and has been identified as a major soil inhabitant^5^,^6^. *Stenotrophomonas* sp. has gathered considerable focus of scientific community due to its important ecological role in bioremediation^7^,^8^, element cycle^9^, plant growth promotion^10^,^11^ and biocontrol of phytopathogens^12^,^13^,^14^,^15^.

Many species of *Stenotrophomonas* have versatile distribution and are found to be widely associated with plants. In relation to agriculture, *Stenotrophomonas* species have the potential to be used as an efficient candidate for plant growth promotion and biocontrol of phytopathogens. Well documented studies have reported that *Stenotrophomonas* species produce plant growth hormone and also generate other factors directly responsible for plant growth promotion^10^. In addition, they also produce antibiotics, volatile organic compounds and fungal cell wall degrading enzymes indicating their key contribution to the biocontrol activity against several soil-borne plant pathogens^6^,^16^,^17^. Furthermore, Dunne *et al*.^15^ elucidated the biocontrol potential of *S. maltophilia* W81 isolated from sugarbeet rhizosphere against damping-off disease causing *Pythium ultimum*. Interestingly, authors also explained production of an extracellular protease as the possible mechanism behind its biocontrol activity. Similarly, in another study Zhang *et al*.^16^ reported that chitinase production by *S. maltophilia* C3 is mainly responsible for its antifungal activity against broad range of phytopathogens. For a long time, *S. maltophilia* has been recognized as opportunist human pathogen but later on, another non-pathogenic species i.e. *S. rhizophila* within the complex of *S. maltophilia* was also identified^18^,^19^. *S. rhizophila* is considered as an important plant growth promoting bacteria and found to be associated with both rhizosphere and phylloplane^20^. This species is differentiated from *S. maltophilia* on the basis of presence of glucosyl glycerol osmolyte (found only in *S. rhizophila*) and hence it exhibits high degree of salt tolerance^21^. Previous report by Egamberdieva *et al*.^22^ demonstrated that *S. rhizophila* DSM14405^T^ mediated growth promotion of various crops in highly salinated soil of Uzbekistan. Congruently, these exciting studies indicate enormous potential applications of *Stenotrophomonas* sp. in agriculture owing to their advantageous interactions with plants.

Remarkably, use of such beneficial microbes as biological inoculants for improving plant growth, biological control of plant diseases and amelioration of stressed environment, offers an environment friendly and sustainable approach^23^. However, their inconsistent performance under field conditions pertaining to several factors limits their practical utility. To overcome such obstacles affecting the translation of biological inoculants into natural environment, the agricultural sector is now exploiting the innovative approach of nanotechnology based strategies^24^,^25^,^26^,^27^. In context to this, several biological agents including microbes and plants are being utilized for biosynthesis of nanoparticles^24^,^28^. Moreover, the biosynthesized nanoparticles are ecofriendly and safe showing robust applications in diverse fields *viz*. pharmaceuticals, cosmetics, medicine, agriculture etc.^29^,^30^. Numerous reports have deciphered successful and efficient applications of biosynthesized nanoparticles for agricultural purposes particularly for plant growth and plant disease management^24^,^26^,^31^,^32^,^33^,^34^,^35^. These exciting studies and above mentioned potential efficiency of *Stenotrophomonas* sp. in agriculture distinctly provide a substantial base for harnessing the nanoparticles synthesis property of this strain and its further utility for agricultural benefits^36^. However, few earlier reports have demonstrated *Stenotrophomonas* sp. isolated from various ecological niches, as a potential candidate for biosynthesis of gold and silver nanoparticles^37^,^38^,^39^. Moreover, these biofabricated nanoparticles also showed promising antibacterial and cytotoxic effects. Interestingly, it is worth mentioning here that so far there have been no reports on the potential role of biosynthesized nanoparticles using this strain in agriculture primarily for plant growth improvement and plant diseases management. In relation to this, the present study investigates the synthesis of silver nanoparticles (AgNPs) by *Stenotrophomonas* sp. BHU-S7 (MTCC-5978) isolated from soil samples from agricultural fields and its efficient role as nanofungicide against a broad range of phytopathogenic fungi. Moreover, we also aim to provide a direct evidence of the inhibitory effect of biosynthesized AgNPs against soil-borne phytopathogen *Sclerotium rolfsii*, causing collar rot of chickpea under greenhouse condition.

## Materials and Methods

### Isolation and identification of bacterial strain

Bacterial strain BHU-S7 was isolated from soil sample collected from agricultural farm of Banaras Hindu University, Varanasi (25°20′ N latitude & 83°01′ E longitude) using the protocol as described earlier^24^. Pure culture of BHU S-7 was maintained on nutrient agar (NA) (HI-MEDIA Laboratories, Bombay, India).

### 16S rDNA sequencing of bacterial strain

The bacterial strain BHU-S7 was identified using 16S rDNA sequence analysis using universal primer pair, forward (5′AGAGTTTGATCCTGGCTCAG3′) and reverse (5′AAGGAGGTGATCCAGCCGCA 3′)^40^. The analysis of partial 16S rDNA sequence was done using BLAST program at National Center for Biotechnology Information (NCBI). The closely related sequences were selected and aligned using Clustal X and further phylogenetic tree was constructed by neighbor-joining method using MEGA 6.0 software.

### AgNPs biosynthesis by bacterial strain BHU-S7 (MTCC 5978)

The AgNPs were biosynthesized by bacterial strain BHU-S7 following the protocol as described earlier by Mishra *et al*.^24^. Aqueous solution of 1 mM AgNO_3_ prepared in sterile distilled water was used for AgNPs synthesis purpose. Briefly, culture supernatant obtained from 48 h grown culture of bacterial strain BHU-S7 in Nutrient Broth (NB) media was treated with 1 mM AgNO_3_ solution and incubated in dark at 30 °C. The biosynthesis of AgNPs was confirmed by visual color change of the solution from yellow to brown. After the completion of synthesis process, AgNPs were collected in the form of pellet by centrifuging the synthesis mixture at 14,000 rpm for 30 min. The obtained pellet was washed several times with sterile distilled water for better removal of any medium components and unconverted Ag^+^ ions as well. The resulting AgNPs in the form of pellet was freeze dried at −80 °C and used for further studies^33^.

### Characterization of biosynthesized AgNPs

The optical characterization of biosynthesized AgNPs was carried out using UV-visible spectroscopy. The absorption spectra of biosynthesized AgNPs solution was monitored by UV–visible spectrophotometer (Perkin Elmer Life and Analytical Sciences, CT, USA) in the range of 300–600 nm. In order to adjust the baseline, nutrient broth media was used as blank. For structural characteristic, X-ray diffraction (XRD) pattern of biosynthesized nanoparticles was performed as described by Singh *et al*.^41^ employing MiniFlex™ II benchtop XRD system (Rigaku Corporation, Tokyo, Japan) operated at 40 kV with Cu-Kα radiation in the range of 2θ = 20°−80°. Further, size and morphology of biosynthesized AgNPs were analysed by scanning electron microscopy (SEM) using JSM 6510LV scanning electron microscope (JEOL, Tokyo, Japan) and transmission electron microscopy (TEM) using JEOL 100/120 kV TEM (JEOL, Tokyo, Japan) operated at accelerating voltage of 20 kV and 200 kV respectively. The Fourier transform infrared (FTIR) spectrum for analyzing functional nature of AgNPs was recorded on Perkin Elmer FT-IR spectrometer Spectrum Two (Perkin Elmer Life and Analytical Sciences, CT, USA) using potassium bromide (KBr) pellets in the ratio of 1:100. The spectrum was recorded in the diffuse reflectance mode at a resolution of 4 cm^−1^ in the range of 400–4000 cm^−1^ wavenumber^42^. For elemental composition, energy dispersive X-ray (EDAX) spectrum was collected using EDAX detector (Oxford Instruments INCAx-sight EDAX spectrometer) equipped with SEM. Furthermore, the thermogravimetric analysis (TGA) was conducted using Pyris1 thermogravimetric analyzer (TGA; Perkin Elmer Life and Analytical Sciences, CT, USA) in order to check thermal stability of biosynthesized AgNPs. For this, sample was subjected to temperature range of 50–700 °C at a heating rate of 10 °C/min under nitrogen atmosphere.

### *In vitro* assay for antifungal activity of biosynthesized AgNPs

The antifungal activity of biosynthesized AgNPs was checked against foliar (*Alternaria alternata, Curvularia lunata* and *Bipolaris sorokiniana*) and soil borne (*Sclerotium rolfsii*) phytopathogens. In case of foliar pathogens, a cavity slide method was used as described by Mishra *et al*.^24^. For this, conidial suspension was harvested in sterile distilled water from pure and freshly grown culture of *A. alternata, C. lunata* and *B. sorokiniana* on Potato Dextrose Agar (PDA) media under aseptic condition. Three experimental sets i.e. control, AgNO_3_ control and AgNPs treated sets were prepared in triplicate for each test fungus. In treated set, the conidial suspension was mixed with 2, 4 and 10 μg/ml of AgNPs and filled in cavity slide. On other side, the control set was maintained by filling the cavity with conidial suspension alone while AgNO_3_ control was maintained by mixing 2, 4 and 10 μg/ml of AgNO_3_ with conidial suspension. The cavity slides from all sets were kept carefully inside the petriplate containing sterilized moist blotting paper. These moist chambers containing cavity slides were incubated at 25 ± 2 °C for 24 h. The slides were examined under light microscope (Nikon DS-fi1, Japan) to observe the inhibitory effect of AgNPs on conidial germination.

Further, inhibitory effect of biosynthesized AgNPs on sclerotia germination of *Sclerotium rolfsii* was investigated by dipping the sclerotia in direct suspension of AgNPs for 12 and 24 h. Later sclerotia were kept on PDA plates for their germination. Sclerotia dipped in sterile distilled water served as control. For more accuracy, sclerotia germination was also checked in soil. For this, control and AgNPs treated sets were prepared in triplicate by placing 25 g garden soil in petriplate. The sclerotia was placed into the soil and drenched with AgNPs suspension maintaining 16% moisture while in control set, soil was drenched with water only. Petriplates were kept in BOD incubator at 25 ± 2 °C for a week.

### Tripartite interaction among *Cicer arietinum, Sclerotium rolfsii* and antifungal biosynthesized AgNPs under Greenhouse conditions

The antifungal efficacy of biosynthesized AgNPs was evaluated against collar rot disease caused by *S. rolfsii* (SR) under greenhouse conditions using chickpea (cv. *Avrodhi*) as a host plant. For conducting greenhouse experiment, plastic pots (5 inch length, 13 cm diameter) were filled with soil (650 g) and 5 chickpea seeds were sown in each pot. For proper seed germination and plant growth, pots were watered to maintain adequate soil moisture. After 15 days of plant growth, following treatments were maintained: (i) unchallenged control (C), (ii) pathogen challenged control (SR control) and (iii) pathogen challenged + AgNPs (SR + AgNPs). Six replicates for each treatment were maintained. The pathogen (*S. rolfsii*) was introduced to the plants by placing one sclerotium adjacent to the collar region of chickpea in each replicate and further covered with moist cotton for maintaining the humidity required for sclerotial germination. For SR + AgNPs treatment, after placing sclerotia on the collar region, AgNPs suspension was sprayed with automizer over this and then covered with absorbant cotton soaked in AgNPs suspension. The infection in collar region by *S. rolfsii* was observed after 24 h of inoculation.

### Chromatographic detection and quantification of phenolic compounds in chickpea

Extraction of phenolic compounds was done following the protocol described by Niranjan *et al*.^43^. For extraction purpose, 1.0 g of plant sample collected (after 24 h of pathogen challenge) in triplicate from each treatment is further processed separately in order to obtain the data in triplicate. Plant sample was crushed with 10 ml of methanol:water:hydrochloric acid solution (5:4:1, v/v) in a mortar and pestle and left overnight in orbital shaker at room temperature. After this, the mixture was filtered through Whatman No. 1 filter paper and plant residue was further extracted twice with same solution for maximum extraction of phenolic compounds. The filtered plant extract was combined together and concentrated upto half of the volume using rotary evaporator (Eyela N–N series, Tokyo, Japan). The residue was further fractionated three times with Ethyl acetate. The collected ethyl acetate fraction was concentrated under reduced pressure on rotary evaporator. The obtained residue was dissolved in 1.0 ml of HPLC grade methanol and filtered through a 0.2 μm membrane filter (Merck). This stock solution was subjected to HPLC for detection and quantification of phenolic compounds.

For HPLC analysis, 20 μl of sample was injected into C-18 HPLC (4 μm, 250 × 4.6 mm, Phenomenex, USA) using HPLC system (Shimadzu, Japan) equipped with LC-10 dual pump and UV detector SPD-10A. The mobile phase used for compounds separation was a linear gradient prepared from acetic acid (1% v/v) in HPLC grade water (pump A) and acetonitrile (pump B), with detection at 254 nm. The flow rate was maintained at 1.0 ml/min. Results were obtained by comparisons with standards using data integrated by Shimadzu class VP series software. Phenolic compounds were detected in sample by comparing retention time (RT) of peaks with that of standard and further quantified by comparing peak area in the sample with that of standard.

### H_2_O_2_ detection in leaves by DAB (3,3′-diaminobenzidine) staining method

Subcellular localization of H_2_O_2_ in chickpea leaves was done using the protocol described by Thordal-Christensen *et al*.^44^. Leaf sample was collected from 6 independent plants of each treatment. DAB staining solution is prepared by dissolving 50 mg of DAB in 45 ml of sterile distilled water and final pH was set to 3 using 0.2 M HCl. After this 0.05% of Tween 20 together with 2.5 ml of 200 mM Na_2_HPO_4_ was added to DAB solution. DAB solution is stored in dark brown bottle as it is light sensitive. For staining purpose, leaf sample was dipped overnight in DAB solution in dark condition at room temperature. After this, leaves were removed from DAB solution and further boiled in bleaching solution containing alcohol and acetic acid (3:1) till leaves become clear (completely devoid of chlorophyll). The boiling step will bleach out the chlorophyll so that brown precipitate formed by reaction of DAB with H_2_O_2_ remains in the leaves. For detection of brown precipitate, leaves were visualized under light microscope (Nikon DS-fi1, Japan).

### Histochemical staining for stem lignification

After 24 h of pathogen challenge, plants were harvested and chickpea stem was used for microscopic investigation of lignification pattern in each treatment using the protocol as described earlier^24^,^45^. Transverse sections of stem were stained in saturated aqueous solution of phloroglucinol in 20% HCl and immediately observed under light microscope for lignification as indicated by red-violet color.

## Results and Discussion

### Identification of BHU-S7

**Figure 1:**
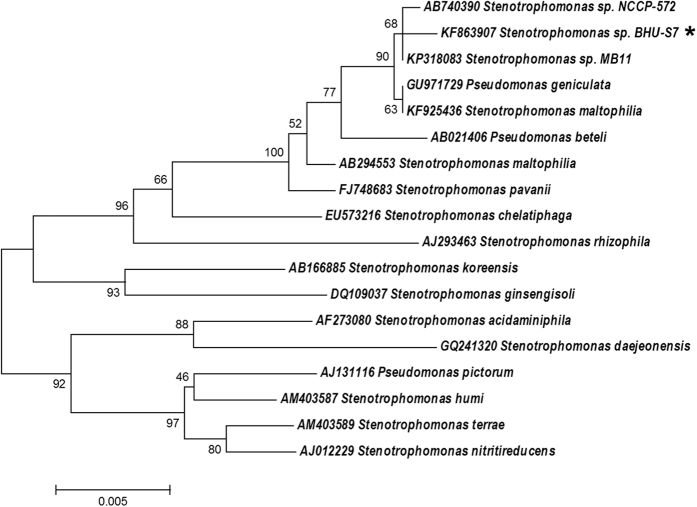
Identification of bacterial strain. Phylogenetic tree of bacterial strain BHU S-7 constructed based on 16s rDNA sequence. It is identified as *Stenotrophomonas* sp. BHU S-7 (as indicated by asterisk). The partial 16S rDNA sequence has been deposited in NCBI GenBank under the accession number KF863907. Bootstrap values are based on 1,000 replications. Bar 0.005 substitutions per site.

The bacterial strain BHU-S7, isolated from agricultural farm soil was identified as *Stenotrophomonas* sp. using 16S rDNA sequencing analysis. The nucleotide sequence of 1445 bp was phylogenetically characterized using BLAST tool of NCBI search. The constructed phylogenetic tree indicated that the bacterial strain BHU-S7 shared a maximum 99% close homology with *Stenotrophomonas* sp. (Fig. 1).

### Nucleotide sequence accession number and MTCC number

The partial nucleotide sequences for 16S rDNA of the bacterial strain BHU S-7 has been submitted to Genbank under the accession number KF863907. Furthermore, pure culture of the bacterial strain has also been deposited at IMTECH, Chandigarh, India with MTCC number 5978.

### Biosynthesis of AgNPs by *Stenotrophomonas* sp. BHU-S7

In this study, *Stenotrophomonas* sp. BHU-S7 isolated from agricultural soil was used for biosynthesis of AgNPs. The visible appearance of dark brown color in culture supernatant upon addition of AgNO_3_ confirms successful extracellular synthesis of AgNPs. In the whole biosynthesis process, the noticeable color change of culture supernatant from yellow to dark brown is due to reduction of silver ions Ag^+^ into Ag° by using active biomolecules present in culture supernatant^46^,^47^. However, no color change was observed in control set where plain growth media was treated with 1 mM AgNO_3_ indicating the functional involvement of bacterial biomolecules in reduction process leading to biosynthesis of AgNPs. The development of brown color in the reaction mixture is mainly attributed to excitation of surface plasmon resonance, a unique optical property of noble metals^48^,^49^. Interestingly, the most widely accepted mechanism for extracellular biosynthesis of AgNPs is *via* extracellular enzymes such as nitrate reductase enzyme present in supernatant that help in transferring the electron to silver ion and hence reduced to AgNPs^47^,^50^.

### Characterization of biosynthesized AgNPs

#### Optical

**Figure 2:**
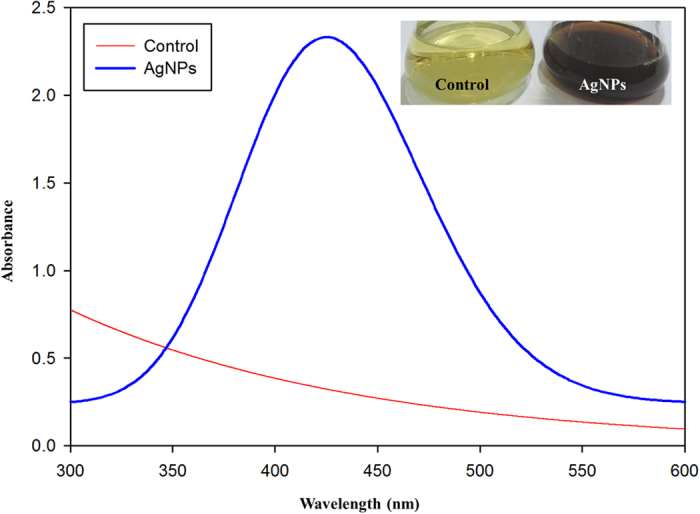
UV–vis absorption spectrum of biosynthesized AgNPs using *Stenotrophomonas* sp. BHU-S7 (blue line) and 1 mM silver nitrate (red line). Inset showing the color change upon formation of silver nanoparticles after treating culture supernatant with 1 mM AgNO_3_.

The unique surface plasmon resonance (SPR) property of noble metals strongly induces the absorption of the incident light and thus can be monitored by Uv-Visible spectroscopy^51^,^52^. Moreover, this SPR band is more prominent for noble metal nanoparticles particularly for AgNPs and AuNPs^53^. Hence, in the present study, the biosynthesis of AgNPs was ascertained by Uv-visible spectroscopy that displayed a strong surface plasmon resonance (SPR) peak at 440 nm (Fig. 2). It is evident from previous reports that AgNPs exhibit UV-visible absorption spectra in the range of 400–500 nm due to Mie scattering^51^. Therefore, presence of characteristic sharp and clear peak at 440 nm indicates successful biosynthesis of AgNPs. While on other side, this distinctive peak was absent in control set signifying no reduction of Ag ions. The reduction process of silver ions and the biosynthesis of stable AgNPs occurred within 24 hour of reaction.

#### Structural

**Figure 3:**
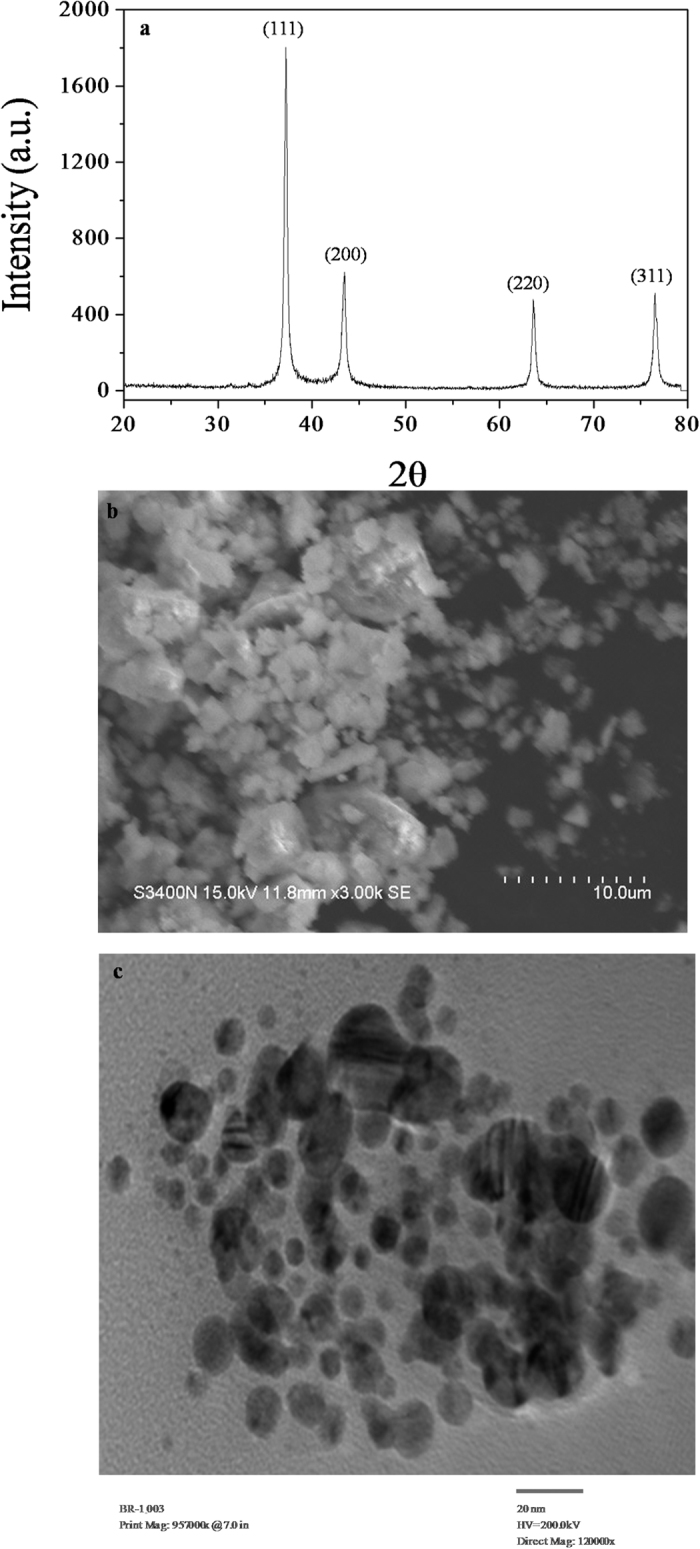
Structural and morphological characterization of biosynthesized AgNPs. (**a**) X-ray diffraction pattern, (**b**) SEM and (**c**) TEM micrographic images showing structure, size and morphology of biosynthesized AgNPs using *Stenotrophomonas* sp. BHU-S7.

The crystallinity of biosynthesized AgNPs was confirmed by X-ray diffraction (XRD) analysis that determines size, lattice and structure of nanoparticles^54^. The diffractogram showed well resolved four diffraction peaks at 2θ angles of 38.39°, 49.25°, 64.10°, and 77.87° which were indexed to the Bragg reflection peaks of (111), (200), (220), and (311) planes of face centered cubic (*fcc*) structures of AgNPs, respectively (Fig. 3a). In this experiment, the XRD data confirmed that the synthesized nanoparticles were crystalline in nature and had a face-centered cubic structure. Further, the average crystallite size (*d*) of biosynthesized AgNPs was calculated following the Debye-Scherrer formula:


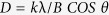


where *k = *0.9 is the shape factor, λ is the X-ray wavelength of Cu Kα radiation (1.54 Å), θ is the Bragg diffraction angle, and B is the full width at half maximum of the (111) plane diffraction peak^55^. The calculated average crystallite size was estimated to be 12.7 nm.

#### Morphological

The morphological investigation of the biosynthesized AgNPs was carried out using SEM and TEM analysis. The Fig. 3b shows the SEM micrograph recorded from dry mass of biosynthesized AgNPs, which was deposited on a carbon tape. The micrograph clearly demonstrated the aggregation of micro particles under the powder form. However, the narrow distribution of AgNPs in the TEM analysis was in accordance with the UV-visible spectral study. The presence of single SPR peak as revealed by UV-visible spectroscopy divulges spherical shape of AgNPs^56^. In the present study, TEM analysis confirms the existence of spherical AgNPs with slight agglomerations at some places that may be due to presence of biological macromolecules on their surfaces (Fig. 3c). Moreover, AgNPs represented variable size ranging from 5 to 30 nm with average mean size estimated to be ~12 nm. However, such variations in size are probably due to the fact that AgNPs were being synthesized at different times^57^.

#### Elemental

**Figure 4:**
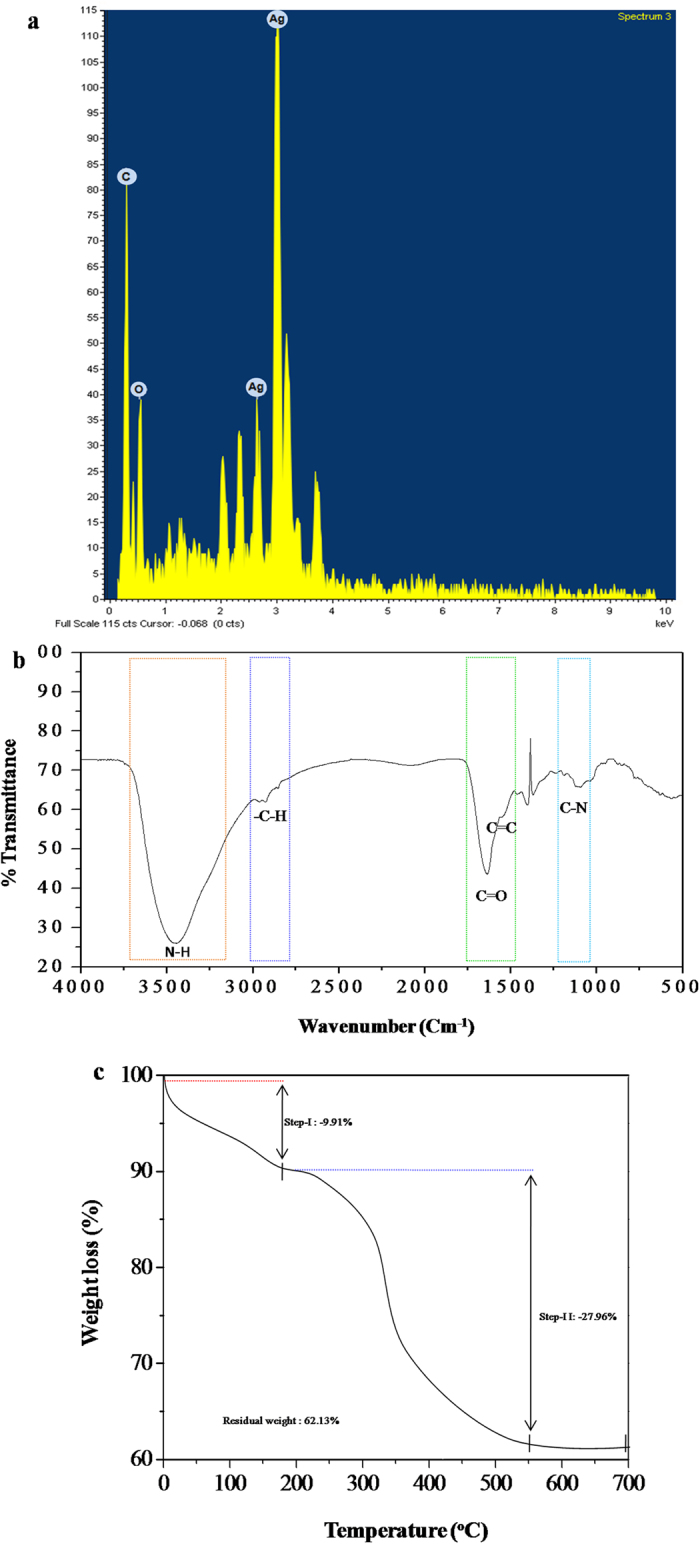
Elemental, functional and thermal characterization of biosynthesized AgNPs. (**a**) EDAX spectrum, (**b**) FTIR spectrum and (**c**) TGA analysis of biosynthesized AgNPs using *Stenotrophomonas* sp. BHU-S7.

The compositional analysis of the biosynthesized AgNPs was carried out by Energy dispersive X-ray (EDAX). The EDAX spectra displayed characteristic signal peak of silver element (Ag) that confirmed the crystallinity and mettalic nature of biosynthesized AgNPs (Fig. 4a). It is worth mentioning here that silver nanocrystallites exhibit distinctive absorption peak at 3 keV because of surface plasmon resonance^58^. Therefore, this data reveal successful synthesis of AgNPs as maximum proportion of total elemental composition is covered by silver followed by carbon (C) and oxygen (O). The occurrences of carbon and oxygen peaks may be due to the biological macromolecules like proteins and enzymes present on the surface of AgNPs.

#### Functional

FTIR analysis was performed in order to gain functional knowledge about the biological macromolecules (that possibly act as reducing/stabilizing agent) present in the culture supernatant of *Stenotrophomonas* sp. BHU-S7. The FTIR spectrum recorded from the dried powder of AgNPs is shown in Fig. 4b. The peaks near 3421 cm^−1^ and 2901 cm^−1^ were assigned to N-H stretching and C–H stretching, respectively. The peak at 1627 cm^−1^ corresponds to amide I, arising due to carbonyl stretch in enzymes and proteins. The broad peak at 1020 to 1250 cm^−1^ corresponds to aliphatic C–N stretching vibrations of the amines. The FTIR spectrum revealed that the carbonyl group of enzymes and proteins has the stronger ability to act as stabilizer by binding to the surface of the AgNPs. This result correlated well with the previous findings^49^,^57^. Altogether, FTIR spectroscopic analysis suggests that the release of extracellular proteins in the culture supernatant of *Stenotrophomonas* sp. BHU-S7 could possibly perform dual functions of synthesis and stabilization of AgNPs in the aqueous reaction medium.

#### Thermal

Thermal stability of the biosynthesized AgNPs was examined by TGA where temperature aided decomposition/desorption behavior of the particles is studied^59^. It has been observed from TGA plot that total dominant weight loss of 37.87% occurred in two steps in the temperature range of 0 °C to 700 °C while 62.13% of residual weight remained in the sample (Fig. 4c). The first step weight loss corresponding to 9.91% was observed at ~100 to 200 °C and the second step weight loss (27.96%) appeared in the temperature range of ~300–450 °C. The first step weight loss can be attributed to evaporation of water molecules adsorbed on the surface of AgNPs whereas second step weight loss is due to desorption of bioorganic components such as proteins and enzymes present in AgNPs sample.

### Proficiency of biosynthesized AgNPs by *Stenotrophomonas* sp. BHU-S7 as nanofungicide against phytopathogens

AgNPs being the most effective and popular antimicrobial agent have capacity to kill nearly 650 types of pathogenic microbes including bacteria, fungi, viruses etc. within 30 minute^60^. The broad spectrum antimicrobial potency of AgNPs is a well-documented fact and has been widely described in many reports^61^,^62^,^63^,^64^,^65^. Similarly, several lines of evidence also describe potential antagonistic activity of AgNPs against diverse range of phytopathogenic fungi^66^,^67^,^68^,^69^. The idea of using biosynthesized AgNPs using biological agents for efficient management of plant diseases has gained noteworthy attention due to the fact that this biosynthesis process involves non-toxic and low cost biomolecules^26^. Moreover, biosynthesized AgNPs being equally competent to those nanoparticles generated by physical and chemicals methods provide safe and environment friendly approach for plant disease management. In context to this, several reports have successfully assessed the strong antifungal activity of biosynthesized AgNPs against phytopathogens^24^,^25^,^26^,^31^,^32^,^70^.

**Figure 5:**
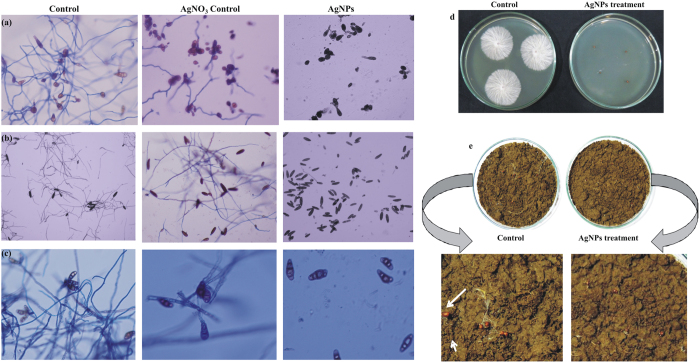
Antifungal efficacy of biosynthesized AgNPs. Inhibitory impact of biosynthesized AgNPs on conidial germination of *Alternaria alternate* (**a**), *Bipolaris sorokiniana* (**b**) and *Curvularia lunata* (**c**). Inhibitory impact of biosynthesized AgNPs on sclerotia germination of *S. rolfsii* on PDA medium (**d**) and in soil (**e**).

As pointed above, the present investigation emphasizes the sturdy impact of AgNPs biosynthesized using agricultural isolate of *Stenotrophomonas* sp. on some devastating plant pathogens such as *A. alternata, C. lunata, B. sorokiniana* and *S. rolfsii*. We examined the probable impact of biosynthesized AgNPs on conidial germination of foliar phytopathogens *A. alternata, C. lunata* and *B. sorokiniana* as this is the main disease causing step. Our data lucidly depict significant complete inhibition in conidial germination at 2, 4 and 10 μg/ml concentration under *in vitro* condition. While, on the contrary 100% conidial germination was observed in control set. In AgNO_3_ control set 20–30% inhibition was reported indicating superiority of AgNPs over AgNO_3_ in controlling plant diseases (Fig. 5a,b,c). The successful conidial germination over leaf surfaces is the main disease determining step leading to development of serious foliar diseases^71^. Hence, AgNPs mediated inhibition in conidial germination of such phytopathogens at very low concentration is of great importance and can be successfully applied for controlling devastating foliar diseases such as leaf spot, sheath blight, spot blotch etc.^24^,^70^. Additionally, we also examined the biocontrol potential of biosynthesized AgNPs against *S. rolfsii*, a soil-borne plant pathogen causing serious diseases on a variety of horticultural and agricultural crops. The efficient management of this soil borne plant pathogen is challenging and difficult as well because of its long term survival in soil due to presence of sclerotia^72^. Moreover, these hard structured sclerotia are well adapted for survival under stressed environment and resistant to physical and chemical degradation due to melanin accumulation in the outer membrane^73^. Therefore, we were more interested in studying the inhibitory impact of biosynthesized AgNPs on sclerotia germination. Our result clearly indicated that AgNPs treatment for 24 h completely reduced the sclerotia germination in PDA medium (Fig. 5d) while there was partial inhibition of sclerotia germination after 12 h treatment with AgNPs (data not shown). Exciting results were also obtained in soil system where noticeable reduction in sclerotia germination occurred after AgNPs treatment while in control set sclerotia started germinating after 24 h of incubation (Fig. 5e).

AgNPs generate highly reactive Ag^+^ ions which is actually responsible for its antimicrobial activity. Indeed, processes such as penetration into microbial cell, disruption of transport system, accumulation of Ag^+^ ions, and production of reactive oxygen species are the definite modes of actions of AgNPs responsible for its cidal effect on microbes^74^,^75^. Hence, it can be hypothesized that inhibitory effect of biosynthesized AgNPs on these phytopathogens is mainly attributed to one of these mechanisms. However, for verifying the exact mechanism a detail study is required.

### Biosynthesized AgNPs mediated suppression of collar rot of chickpea

**Figure 6:**
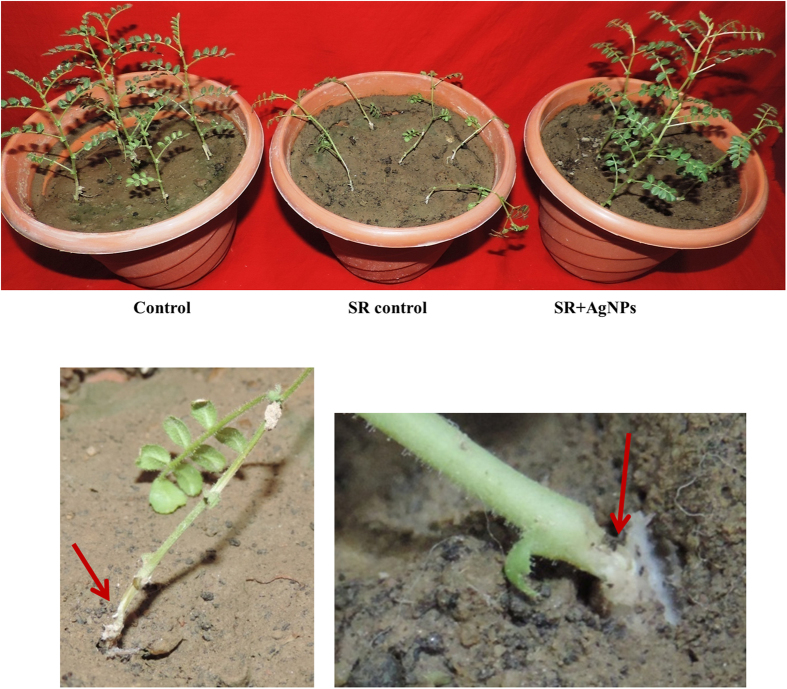
Glasshouse experiment showing successful management of collar rot of chickpea using biosynthesized AgNPs. Red arrow indicates symptoms developed in collar region in *S. rolfsii* challenged chickpea plants (SR control). AgNPs treated challenged (SR + AgNPs) chickpea plants were as healthy as in control.

*S. rolfsii* causes collar rot disease in chickpea leading towards 50–95% mortality at seedling stage. The disease is more prevalent in attacking chickpea seedlings (maximum upto 4–6 weeks after sowing) under favorable condition i.e. high soil moisture and warmer climate^76^. The distinctive disease symptom is characterized by rotting of collar region due to development of whitish mycelial coating followed by yellowing and further collapsing of young seedlings. In the present investigation, greenhouse experiment revealed that AgNPs treatment resulted into suppression of collar rot of chickpea. No characteristic disease symptoms developed at the site of infection i.e. collar region after 24 h of pathogen challenge. Interestingly, chickpea plants looked as green and healthy as in control (without pathogen challenge) set. While in contrary, 100% mortality was observed in pathogen challenged set where plants got collapsed as a result of rotting at collar region after 48 h of pathogen inoculation (Fig. 6). These findings clearly indicated that AgNPs treatment provided protection to chickpea plants against *Sclerotium rolfsii* by hindering sclerotia germination. Our results represent direct evidence of interaction between sclerotia and biosynthesized AgNPs suggesting that reduced sclerotia germination is actually responsible for diminished inoculum potential and disease evidence. Furthermore, it can be hypothesized that such effects of AgNPs might be associated with disruption of sclerotial rind due to Ag^+^ penetration followed by their toxic accumulation inside the cell.

### Defense response induced by phenolics in AgNPs treated chickpea against *S. rolfsii* infection

Phenolic compounds are known to confer disease resistance in plants against pathogen attack. Several mechanisms are involved in phenolic compounds mediated plant defense response that primarily includes building physical/chemical barrier through production of phytoanticipins (preformed) and phytoalexins (induced) and triggering signaling molecules for defense gene induction either locally or systemically^77^,^78^,^79^,^80^,^81^. It is well documented that diverse array of phenolic acids and flavonoids (phenolic acids derivatives) are synthesized in plants through phenylpropanoid pathway, by products of monolignol pathway and breakdown products of lignin^78^,^82^. Redeeming the immense importance of phenolics in plant-microbe interactions, we attempted to corroborate qualitative and quantitative differences in phenolics in response to *S. rolfsii* infection and biosynthesized AgNPs treatment.

**Figure 7:**
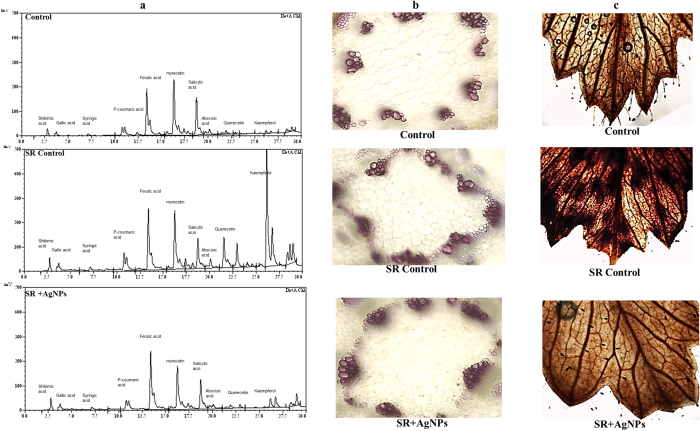
Effect of biosynthesized AgNPs treatment on defense mechanism of chickpea against *S. rolfsii* infection. (**a**) HPLC profiling showing differential pattern in phenolic acids in control, pathogen challenged (SR control) and AgNPs treated challenged (SR + AgNPs) chickpea plants. Effect of different treatment on lignification (**b**) and H_2_O_2_ production (**c**) indicated by DAB staining method.

The phenolic profiles revealed quantitative variations in phenolic compounds among the three treatments (Fig. 7a). The result indicated that most of the phenolic compounds were maximally induced in *S. rolfsii* challenged chickpea plants followed by maximal induction of ferulic acid and myricetin in AgNPs treated challenged (SR + AgNPs) plants. In general, the pattern of phenolic acids induction was observed to be as SR control >SR + AgNPs> Control (Table 1). The maximally induced phenolics in SR challenged plants were found to be shikimic acid (1170 μg/g), gallic acid (53.3 μg/g), syringic acid (118.4 μg/g), p-coumaric acid (232 μg/g), salicylic acid (547 μg/g), abscisic acid (11.7 μg/g), querecetin (46.2 μg/g), kaempferol (324 μg/g). Interestingly, many fold increase in kaempferol (324 μg/g) was reported in pathogen challenged plants as compared to control (10.05 μg/g) and SR + AgNPs (53.5 μg/g) plants. Apart from this, pathogen challenged chickpea plants treated with AgNPs experienced induction of ferulic acid (206 μg/g) and myricetin (121 μg/g) as compared to control and SR control plants. This marked differences in phenolics content among the treatments demonstrated that upon exposure to *S. rolfsii*, the chickpea plants felt stress and exhibited maximum induction of phenolic compounds resulted due to plant’s inherent immunity response in order to fight against the biotic stress. The maximally induced phenolic acids are known to exhibit antioxidant and antimicrobial property^83^,^84^. Alternatively, the direct contact between AgNPs and sclerotia weakened further progression and invasion of pathogen inside the host plant in SR + AgNPs treatment. Consequently, plants sensed relatively lesser stress and hence we observed not much difference in phenolic compounds between control and SR + AgNPs treatment. However, when compared with SR challenged plants, the increased induction of ferulic acid and myricetin was observed in SR + AgNPs treatment. Antioxidant property of ferulic acid and myricetin has been well documented in earlier reports^85^,^86^,^87^,^88^. Furthermore, ferulic acid is known to provide cell wall rigidity by building crosslink between polysaccharides and lignin^89^,^90^,^91^. These reports strongly indicate the potential role played by ferulic acid and myricetin against pathogen attack by making physical barrier and scavenging effect in response to free radicals generated upon exposure to *S. rolfsii*.

### AgNPs mediated impact on lignification and H_2_O_2_ production in chickpea and their role in plant defense

Lignin is an important cell wall component in vascular plants that structurally strengthen and provide rigidity to the cell wall *via* cross linking with polysaccharides^92^,^93^. Lignification of plant tissue has been evidenced as an important mechanism of plant defense against pathogen attack by building a physical barrier to inhibit pathogen invasion inside the host plant cell^94^. Therefore, in the present investigation we attempted to examine the impact of AgNPs treatment on lignification pattern following pathogen attack. Interestingly, maximum lignification was observed in *S. rolfsii* challenged (SR control) chickpea plants followed by moderate lignification in SR + AgNPs treatment and least in control unchallenged plants. This differential response among the treatments resulted due to activation of plant’s inherent immunity response towards pathogen challenge and AgNPs treatment. Our findings point to positive correlation between increased lignification and severe stress condition^95^. Enhanced lignin deposition in cell wall in order to reduce the growth or confine the invading pathogen has been well described in earlier reports^96^,^97^,^98^. Despite of this very well-known fact, the direct impact of AgNPs on plant lignification pattern is a largely unexplored aspect. In relation to this, our study explores the probable impact of AgNPs on plant defense response towards pathogen attack. We evidenced that direct interaction between AgNPs and *S. rolfsii* gave rise to relatively reduced lignin deposition attributed to diminished pathogenicity as compared to SR control. Undoubtedly, it appears that AgNPs potentially exerted its toxic effect on *S. rolfsii* and hence SR + AgNPs treated plants experienced relatively lesser stress and displayed modification in defense response. The almost similar trend of lignification pattern in control and SR + AgNPs treatment revealed immense role of AgNPs in providing protection to chickpea plants against pathogen attack (Fig. 7b).

Apart from this, the strong evidence for AgNPs mediated protection to chickpea plants against *S. rolfsii* infection is well determined by studying *in situ* accumulation of H_2_O_2_ in plants using DAB staining method. In the present study, maximum number of brown spots indicative of H_2_O_2_ production appeared in pathogen challenged (SR control) chickpea leaves. Remarkably, the area and intensity of brown spots were highest due to *S. rolfsii* infection. In contrast to this, unchallenged control plants and SR + AgNPs treated plants displayed similar trend of brown coloration in leaves. It is important to note that the intensity of brown staining was relatively very low in these two treatments as compared to SR control (Fig. 7c). It is evident from earlier reports that H_2_O_2_ being the most stable reactive oxygen species (ROS) is thought to regulate plant development as well as stress response^99^,^100^. Moreover, H_2_O_2_ production in plants is indicative of an early response towards pathogen attack^101^. The results presented here strongly suggest that H_2_O_2_ production increased tremendously following *S. rolfsii* attack in SR control treatment. This elevated level is attributed to early response of chickpea plants to stress condition imposed by *S. rolfsii* by triggering its innate defense response. Plants accumulate higher level of ROS under stressed environment however, at low level they act as signal for triggering other defense response whereas their excessive accumulation lead to oxidative damage to protein, lipid and DNA resulting into plant death^102^,^103^. In relation to this, it can be hypothesized that collapsing of chickpea plants following *S. rolfsii* infection could be due to adverse impact of excessive H_2_O_2_ production on plant growth and other metabolic activity including lipid peroxidation, oxidative damage etc. On other hand, comparatively reduced level of H_2_O_2_ production in SR + AgNPs treated plants provides convincing evidence of important role played by AgNPs in providing protection to chickpea plants against *S. rolfsii*. It indicated that AgNPs treated plants are well equipped to counteract with pathogen challenge by increasing the activity of antioxidant enzymes that reduced the level of ROS and further improved stress tolerance.

## Conclusion

**Figure 8:**
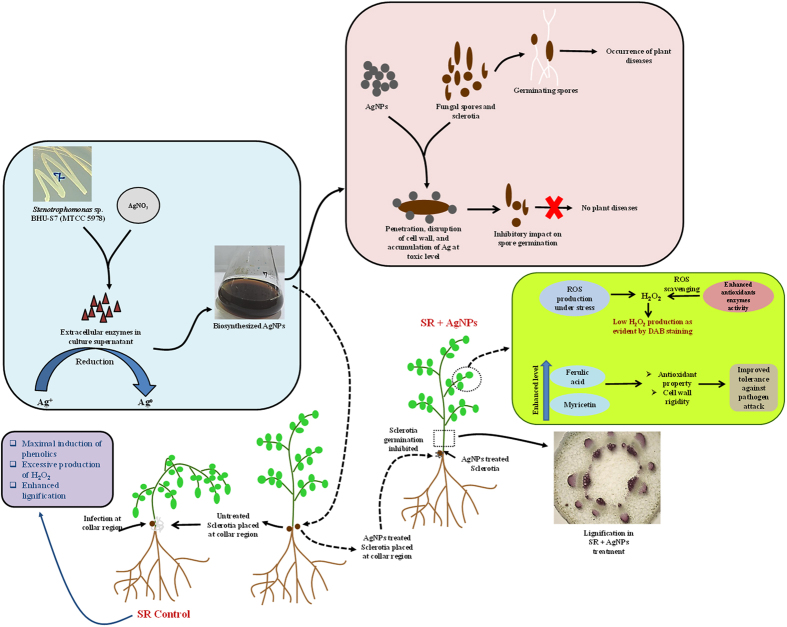
Possible interactions of biosynthesized AgNPs with phytopathogens and chickpea plant. Schematic illustration of the protective impact of biosynthesized AgNPs by *Stenotrophomonas* sp. BHU-S7 (MTCC 5978) on chickpea against *S. rolfsii* infection.

The data presented in this article opens new perspective for efficient agricultural implications of bacterial strain *Stenotrophomonas* sp. that has not been attempted before. This study concerns the broad range antifungal properties of AgNPs biosynthesized using agricultural isolate of *Stenotrophomonas* sp. BHU S-7 (MTCC 5978), against devastating foliar and soil-borne phytopathogens. The biosynthesized AgNPs have been shown to adversely affect pathogenic propagules such as conidia and sclerotia leading to their reduced germination that ultimately diminished the future possibility of disease incidence. Additionally, the biosynthesized AgNPs exhibited successful management of chickpea collar rot disease caused by *S. rolfsii* under greenhouse condition. It is important to note that apart from *in vitro* studies, we made attempt to decipher the proficiency of biosynthesized AgNPs in natural environment for successful practical implication of this work in near future. The biosynthesized AgNPs mediated efficient management of collar rot disease was believed to be operated by many mechanisms. The reduced sclerotia germination, induction of phenolic acids, altered lignification and H_2_O_2_ production was the most reasonable explanation of the protective impact of AgNPs on chickpea against *S. rolfsii* infection (Fig. 8). The almost similar trend of phenolic profiling, stem lignin deposition and H_2_O_2_ production in unchallenged control and SR + AgNPs treatments revealed that AgNPs treated plants experienced lesser stress and are better equipped to cope with pathogen challenge. It also explains that AgNPs treatment helped in reducing the oxidative stress by enhancing the activity of antioxidants enzymes as evident by lowered level of H_2_O_2_ production in comparison to its excessive content in pathogen challenged plants. However, further investigations on studying the level of different antioxidant enzymes are required to support the proposed detail of mechanism. Altogether, the evidence achieved in the present study strengthen the perspective of robust and conceivable applications of AgNPs biosynthesized using greener approach and environment friendly *Stenotrophomonas* sp. BHU-S7 in agricultural sector. Moreover, we have gained new insight about the future possibilities of agricultural isolate of *Stenotrophomonas* sp. particularly for environmental benefits. Rethinking the debate addressing distinction between beneficial and harmful *Stenotrophomonas* strains, we strongly emphasize the selection of agricultural isolates of *Stenotrophomonas* sp. which enable their successful applications in agriculture. This may also be explained by the fact that pathogenic and non-pathogenic *Stenotrophomonas* strains share completely different niches and are likely to be adapted to that environment.

## Additional Information

**How to cite this article:** Mishra, S. *et al*. Potential of biosynthesized silver nanoparticles using *Stenotrophomonas* sp. BHU-S7 (MTCC 5978) for management of soil-borne and foliar phytopathogens. *Sci. Rep.* **7**, 45154; doi: 10.1038/srep45154 (2017).

**Publisher's note:** Springer Nature remains neutral with regard to jurisdictional claims in published maps and institutional affiliations.

## Figures and Tables

**Table 1:** Phenolic acids content (μg/g fresh weight) in chickpea leaves from control, pathogen challenged (SR control) and AgNPs treated challenged (SR + AgNPs) plants.

Retention Time	Phenolics	Control	SR Control	SR + AgNPs
2.5	Shikimic acid	932 ± 28^a^	1170 ± 46^b^	1120 ± 38^b^
3.0	Gallic acid	34 ± 2.1^a^	53.3 ± 3.2^b^	45 ± 2.7^ab^
7.5	Syringic acid	51 ± 2.8^a^	118.4 ± 5.7^b^	64 ± 3.6^a^
11.0	p-Coumaric acid	146 ± 7.8^a^	232 ± 11^b^	162 ± 8.4^a^
13.0	Ferulic acid	170.6 ± 9.6^a^	202 ± 10.4^ab^	206 ± 10.7^ab^
16.0	Myricetin	93 ± 4.3^a^	89 ± 4.9^a^	121 ± 6.8^b^
19.0	Salicylic acid	414 ± 24^a^	547 ± 27^b^	369 ± 21^a^
20	Abscisic acid	8.01 ± 0.6^a^	11.7 ± 0.7^ab^	8.0 ± 0.5^a^
21.5	Querecetin	7.9 ± 0.55^a^	46.2 ± 2.3^b^	14.1 ± 0.8^a^
26.5	Kaempferol	10.05 ± 0.7^a^	324 ± 18^c^	53.5 ± 2.4^b^

Values are expressed as means of three replicates ± SD. Different letters indicate significant differences among treatments within the results taken at the same time interval according to Duncan’s multiple range test at *p* ≤ 0.05.
